# Field and Remote Sensors for Environmental Health and Food Safety Diagnostics: An Open Challenge

**DOI:** 10.3390/bios12050285

**Published:** 2022-04-28

**Authors:** Gerardo Grasso, Daniela Zane, Roberto Dragone

**Affiliations:** Istituto per lo Studio dei Materiali Nanostrutturati Sede Sapienza, Consiglio Nazionale delle Ricerche, P. le Aldo Moro 5, 00185 Rome, Italy; daniela.zane@cnr.it (D.Z.); roberto.dragone@cnr.it (R.D.)

Major foodborne disease outbreaks have clarified the close interconnection and interdependence between the health of humans, animals, and the environment. In different fields, the One Health strategy has come to be recognized by scientists, governments, and consumers as an effective solution for achieving sustainable development [1,2]. The ‘One Health’ strategy is an integrated, unifying approach that aims to sustainably balance and optimize the health of people, animals, and ecosystems. It recognizes the health of humans, domestic and wild animals, plants, and the wider environment (including ecosystems) as closely linked and inter-dependent. This approach aims to promote well-being and address threats to health and ecosystems [3].

Indeed, in the era of translational research, increasing efforts should be devoted to the development of tools for operationalizing the recommendations made by risk assessment. In particular, technological research needs to deepen the understanding of the complex biochemical interactions occurring within environmental matrices and food chains and focus on innovative strategies to improve the diagnostics ability in the field. In fact, no single method or combination of methods used in current monitoring and diagnostic approaches can meet the challenge of modern environmental and food diagnostics. While traditional analytical techniques require sophisticated skills, high costs, and specific/selective methodologies, recent biosensing approaches apply easy analytical protocols and low-cost devices directly on site for the monitoring and/or understanding of risks [4]. Indeed, contamination sources (e.g., industrial, agrozootechnical, household) spill over chemical mixtures (including degradation products) whose ‘net effect’ (i.e., the resultant of additive, antagonistic, and/or synergistic effects) on ecosystems or food production is difficult to predict and assess without such biosensoristic approaches. For example, the evaluation of the net antioxidant power of foods takes into account the resulting effect linked to the coexistence of antagonistic substances such as pro-oxidants/antioxidants. [5].

Whole cell-based or enzyme-based biosensors (biocatalytic sensors) can provide helpful information about the bioactivity and exposure effects of substances and mixtures on cellular functions, thus supporting the evaluation and monitoring of bioavailability and toxicity. This can be very useful if, e.g., chemical hazards are not known a priori and/or preliminary screening is required to guide confirmatory sophisticated analyses. Biosensors that use a biological mediator (e.g., phage, aptamers, antibodies in relative stable and selective complexes) towards a target analyte (the so-called ‘affinity-based biosensors’) are very promising for use in diagnostic applications. Scalable technology that is able to operate in situ in real time will serve as an early alert and intervention in natural contexts, including food production systems and related corrective actions.

New and innovative sensor-based technologies can provide new opportunities to explore and protect agro-zootechnical productions. Biosensors can be adapted for application in several steps of food production, from pre-harvest agriculture to intelligent food packaging [6]. Recently, interest in the use of sensor-based systems in dairy farms has grown [7,8]. Biosensor-based technologies can improve many aspects of livestock production, from herd management, animal welfare, and health monitoring to the quality and safety of animal source foods [9]. So-called ‘precision livestock farming’ points to a herd management system based on a ‘per animal’ approach; the incorporation of new and upcoming technologies and tools and the continuous automatic real-time monitoring and control of production/reproduction, animal health, and welfare; and the monitoring of the environmental impact of livestock production [10]. If properly applied, precision livestock farming can potentially have a positive impact on dairy farming, increasing the efficiency and sustainability of farming and livestock production, improving animals’ welfare, and supporting traceability across the entire food supply chain [1,7].

Recent improvements made in (nano) material science, (nano) engineering, networking, communication technologies, and artificial intelligence technologies have paved the way for the new concept of ‘smart sensors’. A further development, still little explored, concerns the use of biosensoristic devices in combination with other sensoristic technologies (terrestrial/proximal chemosensors, physical sensors, and remote sensing systems).

In recent years, remote sensing techniques using multi/hyperspectral imaging technologies have proved their effectiveness as affordable and practical supports for more traditional sensor-based methodologies. The combined use of hyper/multispectral sensors with different levels of spectral discrimination (both in terms of the number and position of bands) and the analysis of images acquired from satellite platforms and/or unmanned aerial vehicles (a.k.a. drones) allows for the acquisition of sets of data that can be useful for defining the spectral signature of vegetation, water, surfaces, and soils. The integration of remote sensing data with those derived from field studies (from in situ measurements to laboratory studies) can really be helpful to fully understand complex spatial and temporal alteration in ecosystems [11].

However, the promising dialogue that occurs between multisensoristic technologies will require the implementation of both communication protocols and tools for data handling and analysis. For these purposes, the recent evolution of enabling technologies and connectivity models should speed up the progress of wireless sensor networks (WSNs) into the internet of things (IoT). Such advances can lead to the creation of improved sensor networks for environmental monitoring and Smart Agriculture applications [12,13]. In this context, the (next) tough challenge is the proper handling of the exponential growth of data generated by the increasing number of sensors. This will require the use of improved data processing techniques. The use of artificial intelligence (AI) technologies can certainly help to solve such complexity, providing new insights into data processing and data mining for real-world sensing systems [14]. The AI technology-mediated integration of different raw data generated from arrays of multiple types of sensors (chemical, physical, biological sensors) is particularly interesting. The analysis of numerous parameters can lead to the extraction of meaningful information from complex datasets and the identification of new markers that could be useful for the multitemporal monitoring and diagnostics of natural contexts or food production systems. This integration opens enormous potential for overcoming the limits of traditional environmental monitoring and diagnostic techniques. It expands the grid of parameters up to the definition of a ‘fingerprint’ monitorable over time, leading to a more complete characterization of matrices.

This Special Issue collects research papers and reviews on field and remote sensing-based devices applied in the field of Environmental Health and Food Safety Diagnostics. Works with an interdisciplinary character that encourage the innovation of the application of sensors are welcome as well.

## Data Availability

Not applicable.
